# Neutrophils in the initiation and resolution of acute pulmonary inflammation: understanding biological function and therapeutic potential

**DOI:** 10.1002/path.5221

**Published:** 2019-02-15

**Authors:** Philippe MD Potey, Adriano G Rossi, Christopher D Lucas, David A Dorward

**Affiliations:** ^1^ The University of Edinburgh Centre for Inflammation Research, Queen's Medical Research Institute University of Edinburgh Edinburgh UK

**Keywords:** neutrophil, ARDS, apoptosis, inflammation, neutrophil extracellular trap, chemokine, interleukin, leukotriene, DAMP, PAMP, toll‐like receptor, reactive oxygen species

## Abstract

Acute respiratory distress syndrome (ARDS) is the often fatal sequelae of a broad range of precipitating conditions. Despite decades of intensive research and clinical trials there remain no therapies in routine clinical practice that target the dysregulated and overwhelming inflammatory response that characterises ARDS. Neutrophils play a central role in the initiation, propagation and resolution of this complex inflammatory environment by migrating into the lung and executing a variety of pro‐inflammatory functions. These include degranulation with liberation of bactericidal proteins, release of cytokines and reactive oxygen species as well as production of neutrophil extracellular traps. Although these functions are advantageous in clearing bacterial infection, the consequence of associated tissue damage, the contribution to worsening acute inflammation and prolonged neutrophil lifespan at sites of inflammation are deleterious. In this review, the importance of the neutrophil will be considered, together with discussion of recent advances in understanding neutrophil function and the factors that influence them throughout the phases of inflammation in ARDS. From a better understanding of neutrophils in this context, potential therapeutic targets are identified and discussed. © 2018 The Authors. *The Journal of Pathology* published by John Wiley & Sons Ltd on behalf of Pathological Society of Great Britain and Ireland.

## Introduction

Acute respiratory distress syndrome (ARDS) is the often fatal final sequalae to a broad range of direct and indirect pulmonary insults that include both infective and sterile aetiologies such as pneumonia, aspiration of gastric contents, sepsis, acute hepatic failure and acute pancreatitis. ARDS is defined by an acute onset of respiratory symptoms; profound systemic hypoxaemia; diffuse, bilateral infiltrates on chest X‐ray and the exclusion of cardiac failure or fluid overload as a precipitant 1. Despite decades of intensive research, the mortality rate for ARDS remains approximately 40%, with no effective pharmacological therapies in routine clinical practice 2. The failure to translate a large number of promising therapeutic agents from preclinical studies is well described 3. Challenges arise when attempting to develop drugs that span the diverse and heterogenous conditions that precipitate ARDS, the differences in the inflammatory phenotypes and underlying genomic variation within this patient population, as well as the difficulties in the translation of observations in animal models into human inflammatory disease 3. Distinct from interindividual variation is also the complexity of redundancy and dysregulation of the inflammatory environment that characterises ARDS. Despite these challenges, the need to develop novel therapeutics is pressing.

ARDS is characterised by an overwhelming, dysregulated and self‐perpetuating pro‐inflammatory environment; there is a significant increase in a range of pro‐inflammatory mediators accompanied by rapid recruitment of neutrophils into the alveolar space, endothelial injury and dysfunction, platelet aggregation and microthrombus formation, interstitial and alveolar oedema, alveolar epithelial cell death and macrophage activation 4. Diffuse alveolar damage is the typical histological hallmark of this exudative phase, although the histological appearances can be very variable between individuals who have died from severe ARDS 5. Following alveolar damage, there is a proliferative phase with resolution of pulmonary oedema, type II alveolar cell hyperplasia, early collagen deposition and release of pro‐resolving mediators, including lipoxins and resolvins 6, 7. Although inflammation and injury completely resolve to leave no clinical, radiological or physiological impairment in some individuals, there remains a substantial cohort who subsequently develop diffuse pulmonary fibrosis and chronic lung disease 8.

Within this inflammatory milieu there are multiple cell types with direct roles in disease pathogenesis, including macrophages, epithelial and endothelial cells. There is, however, an established body of literature that implicates the neutrophil as central to driving this inflammatory state 9, 10. Increased neutrophil numbers, the presence of neutrophil‐derived proteases and the chemotactic factors that drive neutrophil recruitment are associated with increased disease severity and higher mortality rates 9, 11. Similarly, neutrophil depletion, inhibition of key chemokines and signalling molecules, or acceleration of apoptosis to shorten neutrophil lifespan, results in improvement in oxygenation, reduction in inflammation and accelerated inflammation resolution in preclinical models 12, 13, 14, 15. To date, however, clinical trials targeting neutrophil function in ARDS have failed to show benefit in overall survival 3, 16.

Although much has been written on the detailed mechanisms of neutrophil migration and function in inflammation 17, 18, 19, this review focuses on those observations that have been demonstrated within the context of ARDS and preclinical models of acute lung injury. In doing so we hope to provide a focus on those pathological mechanisms that are of potential clinical relevance and may therefore represent therapeutic targets of the future (Table 1).

**Table 1:** Neutrophil‐related mediators in ARDS, preclinical observations and associated therapeutics

		Preclinical observations	Human ARDS	Therapeutic potential	Therapeutics	Reference
PAMPs		Neutrophil recruitment and activation	Unknown	TLR1, TLR4, TLR5 antagonists	Not clinically tested	23, 143
DAMPs	Mitochondrial formylated peptides	↑ neutrophil migration and inflammation	↑ in blood and BALF	FPR1 antagonists	Not clinically tested	31
	Mitochondrial DNA	↑ neutrophil migration and inflammation	↑ in blood and BALF	TLR9 antagonists	Not clinically tested	30
	HMGB1	↑ neutrophils and inflammation	↑ in blood	Metformin	No increase in survival	132, 133, 144
Chemokines/cytokines	CXCL5	Neutrophil chemotaxis	↑ in BALF	CXCL5 antibody CXCR2 antagonist	Not clinically tested	145
	CXCL8	Neutrophil chemotaxis	↑ in blood and BALF	CXCL8 antibody CXCR1, CXCR2 antagonist	Allogeneic adipose‐derived mesenchymal stem cells – no effect	36, 38, 146
	CCL2	Neutrophil chemotaxis	↑ in BALF	CCL2 antibody CCR2 antagonist	Not clinically tested	39
	CCL7	Neutrophil chemotaxis	↑ in BALF	CCL7 antibody CCR1, CCR2, CCR3 antagonist	Not clinically tested	39
	LTB_4_	Neutrophil chemotaxis	↑ in blood and BALF	LTB_4_ antibody BLT2 antagonist	Not clinically tested	147, 148
	C5a	Neutrophil chemotaxis	↑ in BALF	Anti‐DBP	Not clinically tested	149
	TNF	Pro‐inflammatory response Microvascular plasma protein leakage	↑ in blood and BALF	TNF antibody TNF‐RA	Not clinically tested TNFR1 antibody – ↓ pulmonary neutrophilia, inflammatory cytokine release, endothelial injury	41, 43, 50, 150
	IL‐1β	Pro‐inflammatory response	↑ in blood and BALF	IL‐1β antibody IL‐1RA	Not clinically tested	50, 150
	TNF receptor	Neutralises TNF imbalance	↑ in BALF	Administration for TNF neutralisation	Not clinically tested	50
	IL‐1RA	Neutralises IL‐1β imbalance	↑ in BALF	Administration for IL‐1β neutralisation	Not clinically tested	50
Selectins	L‐selectin	Aids in neutrophil migration	Soluble form ↓ in blood	L‐selectin antibody	Not clinically tested	57, 151
	E‐selectin	Aids in neutrophil migration	Soluble form ↑ in blood	E‐selectin antibody	Not clinically tested	57
	P‐selectin	Aids in neutrophil migration	Soluble form ↑ in blood	P‐selectin antibody	Not clinically tested	57, 66
Integrins	β2 integrin	Aids in neutrophil migration	↑ sICAM‐1 in blood and BALF	β2 integrin antibody	Not clinically tested	62, 65
NETs		Lung injury	↑ in BALF	DNAse I	Inhaled DNAse I – phase III	68, 70, 152
Granule proteins	NE	Lung injury	↑ in blood and BALF	Anti‐elastase therapies	No increase in survival	72, 79, 153, 154
	Elafin	NE inhibitor	↓ in blood	Administration	Not clinically tested	154
	MMP‐1	Lung injury	↑ in BALF	Inhibit with TIMP	Nebulised hypertonic saline – phase I	83, 86, 88, 155
	MMP‐2	Lung injury	↑ in BALF	Inhibit with TIMP	Nebulised hypertonic saline – phase I	83, 86, 88, 155
	MMP‐3	Lung injury	↑ in BALF	Inhibit with TIMP	Nebulised hypertonic saline – phase I	83, 86, 88, 155
	MMP‐8	Lung injury	↑ in blood and BALF	Inhibit with TIMP	Nebulised hypertonic saline – phase I	83, 86, 88, 155, 156
	MMP‐9	Lung injury	↑ in BALF	Inhibit with TIMP	Nebulised hypertonic saline – phase I	83, 86, 88, 155
	TIMP‐1	Inhibits MMPs	↑ in blood and BALF	Administration	Not clinically tested	83, 156
	HBP	Vascular leakage	↑ in blood	Simvastatin reduced serum HBP	Simvastatin ↑ survival in hyper‐inflammatory subphenotype	89, 90, 92, 93
	α‐defensin	Lung injury	↑ in blood and BALF	Inhibit	Not clinically tested	95
	β‐defensin	Inhibits neutrophil apoptosis	Unknown	Inhibit	Not clinically tested	96
	LL‐37	Neutrophil activation	↑ in BALF	Inhibit	Not clinically tested	97
ROS	H_2_O_2_	Lung tissue damage Release pro‐inflammatory mediators Immune cells recruitment	↑ in breath condensate	Neutralising with antioxidants Pan‐PI3K inhibitor	Inhaled carbon monoxide – phase I	99, 112, 157, 158
	Glutathione	Neutralises H_2_O_2_	↑ oxidised glutathione in BALF	Intravenous administration	No effect	101, 102
Apoptosis	PAI‐1	↓ neutrophil apoptosis	↑ in BALF	PAI‐1 antagonist	Not clinically tested	159
	Mcl‐1	↓ neutrophil apoptosis	↑ in ARDS neutrophils	CDK inhibitor	Not clinically tested	111

DBP, vitamin D‐binding protein; H_2_O_2_, hydrogen peroxide; BLT2, leukotriene B_4_ receptor 2; PAI, plasminogen activator inhibitor.

## Neutrophil recruitment and function

**Figure 1:**
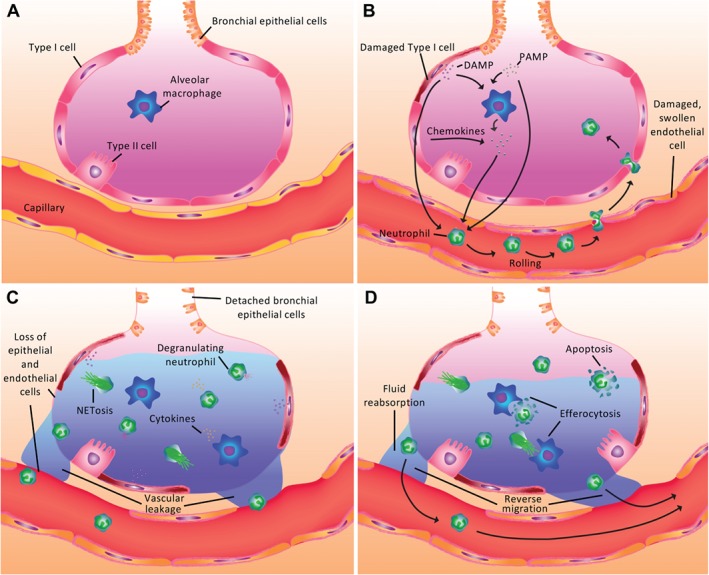
Initiation and resolution of neutrophil‐mediated inflammation in ARDS. (A) The healthy alveolar unit facilitates rapid gas transfer with the presence of resident alveolar macrophages providing rapid response to pulmonary infection and injury. (B) Following infection and/or tissue injury, release of PAMPs and/or DAMPs directly induces neutrophil recruitment into the alveolar space in addition to a range of chemokines and mediators secreted by macrophages and epithelial cells. (C) Neutrophils exert multiple pro‐inflammatory functions with the release of ROS, proteases, NETs and cytokines, as well as phagocytosis of bacteria. This is accompanied by accumulation of oedema within the alveolus, endothelial dysfunction and epithelial cell death. (D) Resolution of inflammation occurs through neutrophil apoptosis and macrophage clearance of apoptotic cells (efferocytosis) and inflammatory debris. The role of neutrophil reverse migration remains to be fully characterised in ARDS.

The recruitment of neutrophils to the lung makes them a key factor in the pathogenesis of ARDS. In response to inflammatory mediators, either originating from the lung or distant organ injury, circulating neutrophils become primed and alter their cytoskeletal architecture with retention in the pulmonary capillary bed. They then migrate out of postcapillary venules across the endothelium, through the interstitium and epithelium and into alveoli, with associated local tissue dysfunction and destruction due to release of histotoxic mediators, such as neutrophil extracellular traps (NETs), reactive oxygen species (ROS) and proteases (Figure 1). This induction of epithelial and endothelial injury contributes to the development of alveolar oedema and hypoxaemia, as well as exacerbating the pro‐inflammatory state. It should be recognised that neutrophil migration into the lung without concomitant activation does not induce tissue injury 13. However, there are conflicting models with regards to the mechanisms by which initial neutrophil activation occurs. It has been proposed that activation of the intravascular immune system, through an increase in circulating pro‐inflammatory mediators, results in neutrophil priming, adhesion and/or trapping in lung capillaries. Subsequent migration along a variety of chemotactic gradients into the lung parenchyma therefore results in secondary lung injury 20. The alternative hypothesis is that the release of pro‐inflammatory mediators by alveolar macrophages plays a vital role in the initiation of inflammation 21, triggering an inflammatory cascade by activating surrounding tissues and resulting in chemotaxis of inflammatory cells, such as neutrophils, to the airways 21. It is likely that the exact mode of initial neutrophil recruitment and activation varies depending on the inciting stimulus and whether this is intrapulmonary or systemic. However, the end result in both cases is the recruitment of neutrophils to the lung, resulting in tissue injury.

## Pathogen‐associated molecular patterns (PAMPs) and damage‐associated molecular patterns (DAMPs)

Both sterile and infective tissue injury result in neutrophil recruitment into the lung through complementary mechanisms. In the context of infection, PAMPs including lipopolysaccharide (LPS), lipoteichoic acid, DNA, RNA and proteins such as formylated peptides are released and recognised by the immune system 22. PAMPs can bind to, and are sensed by, a variety of pathogen recognition receptors, including Toll‐like receptors (TLRs) and Nod‐like receptors 23. Pathogen recognition receptors and their downstream signalling pathways drive chemotaxis, as well as priming and activating both intravascular and transmigrated neutrophils in order to fulfil their bactericidal functions 22, 24. TLRs play an important role in regulating the response to pro‐inflammatory mediators and are rapidly upregulated in mouse models of sepsis‐related acute lung injury 25. In early sepsis‐related ARDS, downregulation of TLR1, TLR4 and TLR5 transcripts in mononuclear cells correlates with increased survival 23, whereas in a pulmonary contusion mouse model of lung injury, alveolar neutrophil recruitment is TLR4/MyD88‐dependent 26. Similarly, TLR4 deficiency is associated with a reduction in sterile pulmonary inflammation and more rapid resolution of injury through alterations in downstream synthesis of cysteinyl leukotrienes and subsequent induction of SOCS3 within the lung. In this context, a reduction in TLR4‐mediated oxidative stress was observed but, surprisingly, alveolar neutrophil numbers were increased 27. Although this suggests an important role for TLRs in acute lung injury, the functional importance of neutrophils in this model is limited, thereby serving to emphasise that understanding the neutrophil‐mediated TLR function in ARDS requires further investigation.

Sterile tissue injury, either in the context of direct injury to lung parenchyma or distant organ injury, results in necrotic cell death with the release of a range of DAMPs into the extracellular environment. These DAMPs serve to induce a pro‐inflammatory response, which drives neutrophil recruitment into the lung 25, 27. A number of DAMPs have been described in ARDS, including high mobility group box 1 (HMGB1), heat shock proteins 60 and 72, hyaluronan and a range of mitochondrial‐derived factors, including DNA, formylated peptides and cardiolipin 28. Due to common evolutionary ancestry and relative structural and sequence homology with bacteria, it appears that these mitochondrial factors play an important role in driving the development of neutrophil‐mediated lung injury. Mitochondrial DNA is elevated in patients with ARDS and, through interaction with endosomal TLR9, mediates neutrophil recruitment 29. Importantly, it has also been shown to be a predictive biomarker of mortality in patients in intensive care, including those with ARDS, and therefore further studies with regards to both its role as a clinically significant predictive biomarker in ARDS and as a therapeutic target are needed.

Mitochondrial formylated peptides play a crucial role in neutrophil recruitment in ARDS, as well as altering epithelial and endothelial cell function 29, 30, 31. Elevated levels of these peptides are found in bronchoalveolar lavage fluid (BALF) and serum of ARDS patients 31. The importance of formyl peptide receptor 1 (FPR1, the cognate receptor for formylated peptides) in influencing acute inflammation is well established 32. Genetic deletion of *Fpr1* in mice is associated with reduced survival in infection but an attenuated inflammatory response in the context of sterile tissue injury 29, 31, 33. FPR1, a G‐protein coupled receptor, activates a variety of intracellular signalling pathways, including PI3K, MAPKs and Akt pathways 34. This serves to directly alter neutrophil migration, ROS production, degranulation and transcriptional activity 32. In sterile lung injury in mice, neutrophil chemotaxis, together with other indices of pulmonary inflammation, were diminished in *Fpr1* ^−/−^ mice or in the presence of an FPR1 antagonist delivered either prior to or following acid‐induced lung injury. This suggests that FPR1 may represent a therapeutic target in sterile ARDS but, as with many other therapies targeting neutrophil function, the challenge of concurrent infection needs to be addressed 31.

## Chemokines and cytokines

Chemokines, a family of chemotactic cytokines, play a crucial role in neutrophil migration to sites of inflammation 35. CXC chemokines, in particular CXCL8 (IL‐8), play an important role in neutrophil chemotaxis in ARDS, with elevated levels associated with poor disease prognosis and increased severity and mortality 36, 37, 38. Produced by local immune cells and epithelial cells, CXCL8 is not the only chemokine responsible for the recruitment of neutrophils to the lung, as blockade results in only partial reduction in alveolar neutrophil number 13, 35. CCL2 and CCL7 are also elevated in the BALF of both LPS‐challenged volunteers and ARDS patients while neutralising either chemokine reduces neutrophil chemotactic responses *in vitro* 39. Interestingly, CCL2 and CCL7 also potentiate the activity of CXCL8, suggesting that a synergistic activity between these chemokines drives neutrophil recruitment in ARDS. The CXCL8 receptor, CXCR1, is more highly expressed on circulating neutrophils from ARDS patients relative to the CCL2/7 receptors CCR1, CCR2 and CCR3 39. However, a significant increase in neutrophil CCR2 expression in BALF has been observed, with the authors postulating that this confers an increased sensitivity to cognate ligands CCL2 and CCL7 in the alveolar space and therefore suggesting an important role in neutrophil chemotaxis within the lung. Other chemokines, including CXCL5, and mediators such as C5a and leukotriene B_4_ (LTB_4_), have also been shown to have a role in driving neutrophil chemotaxis in ARDS 40.

Although most cytokines are produced by other cell types, neutrophils also secrete a range of cytokines that potentiate the inflammatory response. These include TNF, which has been associated with microvascular plasma protein leakage 41, and IL‐1β, which potentiates the pro‐inflammatory cycle by inducing further cytokine and chemokine release and thereby recruiting more neutrophils to the lung 42. Furthermore, antibody‐mediated inhibition of TNFR1 reduced alveolar neutrophil recruitment, inflammatory cytokine release and biomarkers of endothelial injury in BALF and serum samples in experimental acute lung injury. As a result, inhibiting TNFR1 could be considered as a potential option in the treatment of ARDS 43.

After a non‐pulmonary acute injury, such as traumatic brain injury, burn injury or sepsis, mediators including IL‐1β, IL‐6, CXCL8, IL‐18 and TNF, as well as a variety of DAMPs, are released into the systemic circulation 23, 36, 44, 45, 46, 47. For example, intravascular neutrophil priming and activation, as part of a systemic inflammatory response syndrome that occurs following traumatic brain injury, results in neutrophil migration into the lung 47 and other organs, including the liver and kidney 48, 49, inducing tissue injury and dysfunction. Similarly, the pro‐inflammatory cytokines TNF and IL‐1β were found to be elevated in the BALF of ARDS patients alongside the natural antagonists IL‐1RA and soluble TNF receptors 50. It appears, however, that an imbalance between agonists and antagonists exists, which drives the acute phase inflammatory response 51.

At present, there have been no specific clinical trials investigating the pharmacological manipulation of the majority of chemokines, although steroids effecting their function through suppression of chemokine/cytokine axis have been proposed. Current clinical trial data on the use of steroids in ARDS are mixed and there is no definitive evidence for improved survival and in some cases has been found to worsen outcome. Although improved effects on ventilator‐free days have been described, the return to mechanical ventilation is increased among those receiving steroids, as are significant side‐effects, including neuromyopathy and hyperglycaemia 52, 53, 54, 55, 56.

## Selectins and integrins

In order for neutrophils to enter the alveolar space, selectins play an important role in initiating the process of neutrophil tethering and rolling along the endothelial surface. L‐selectin, one such adhesion molecule present on neutrophils, has been found (in its soluble form) to be reduced in the plasma of ARDS patients, which directly correlated to ventilation requirements, degree of respiratory failure and mortality 57, 58. Conversely, elevated plasma levels of E‐selectin and P‐selectin, expressed by endothelial cells, correlated with increased mortality 58, 59, 60. Most recently, through genome‐wide association studies, three non‐synonymous SNPs in the selectin P ligand gene (*SELPLG*) have been identified to be associated with sepsis‐related ARDS 61. PSGL1 (the encoded protein) acts as an important ligand for both L‐selectin and P‐selectin. In LPS‐induced lung injury, inflammation is attenuated in *Selplg* ^−/−^ mice, while an inhibitory antibody to PSGL‐1 also limits inflammation in LPS‐ and ventilator‐induced lung injury models 61. The exact mechanisms through which the SELPLG SNPs exert their functional effect is not known. It was postulated that alteration in amino acid sequence may result in altered P‐selectin binding affinity and therefore alter neutrophil rolling 61.

Once tethering and rolling are initiated, integrins play a role in slowing down and immobilising neutrophils to allow transendothelial migration and activation 62. Surprisingly, neutralising antibodies to the β2 integrin CD18 in the context of sterile lung inflammation results in increased alveolar neutrophils but a reduction in neutrophil‐mediated pulmonary injury, suggesting that its predominant role is in neutrophil activation rather than chemotaxis 13. β2 integrins on the surface of activated neutrophils induce heparin‐binding protein (HBP) release through PI3K‐dependent signalling 63. Antibody‐mediated blockade of β2 integrin function resulted in lower levels of circulating HBP and a subsequent reduction in pulmonary oedema, which the authors proposed was principally through a reduction in vascular leak and endothelial dysfunction 46. The β2 integrin binds to ICAM‐1 on endothelial cells to aid in neutrophil transmigration 64. Soluble ICAM‐1 is elevated in ARDS patients and its inhibition reduces sterile lung injury in mice 65, 66.

## NETosis

NETosis, the process through which neutrophils release extracellular DNA in order to trap and contain bacteria, is an important defence mechanism against invading pathogens. Increased NET production has recently been associated with increased ARDS severity 67, 68. Lefrançais *et al* 68 demonstrated that circulating neutrophils from ARDS patients produce significantly more NETs upon phorbol myristate acetate stimulation than those from healthy donors. As NETs contain and can release neutrophil elastases (NE), myeloperoxidase, DNA and histones, they can also potentiate the tissue damage observed in ARDS, in part through cytotoxic effects on epithelial and endothelial cells 69. Reducing NETs either by intratracheal DNase I treatment or the partial deficiency of protein arginine deiminase 4 (*PAD4* ^*+/−*^; a protein involved in the projection of NETs) increased survival in a mouse model of severe bacterial pneumonia/acute lung injury 68. Although partial deficiency of PAD4 reduces lung injury, complete knock out increases bacterial burden. This suggests that a NET balance is necessary and that the potential deleterious or beneficial effects of NETs in ARDS may relate to the presence of microbial infection 68. Furthermore, a phase III clinical trial is currently investigating the effectiveness of inhaled dornase alfa recombinant human DNAse 1, in reducing the incidence of moderate to severe ARDS in severe trauma patients through accelerated degradation of extracellular DNA, including NETs (Table 1) 70.

## Granule proteins

The release of various granule proteins, including elastases, matrix metalloproteinases (MMP) and cationic polypeptides, has been associated with the propagation of ARDS 71. NE are implicated in lung injury, although it is unclear whether the damage is principally to endothelial or epithelial cells or as a result of degradation of the alveolar basement membrane 72, 73. Plasma levels of NE and the endogenous proteinase inhibitor elafin are predictors of ARDS mortality 74, whereas inhibition of NE reduces lung injury in various animal models 75, 76, 77, 78, 79. Mice deficient in NE are more susceptible to Gram‐negative bacteria, suggesting that NE are required for adequate host defence against invading pathogens and complete inhibition of NE can be harmful 80. Despite data from preclinical models, sivelestat, a selective NE inhibitor, did not alter 28‐day mortality in a number of clinical trials (Table 1) 81. Although alteration in oxygenation has been observed, the small sample sizes of the majority of clinical trials and heterogenous patient populations potentially mask any benefit in subgroups of patients with ARDS 81. It is difficult to separate challenges in clinical trial design from limitations of biological importance in this context. Therefore further study is required to clarify both aspects of this problem.

MMPs are zinc‐dependent endopeptidases with numerous biological functions, such as tissue remodelling, wound healing and angiogenesis 82. Fligiel and colleagues 83 investigated numerous MMPs and their natural antagonists, tissue inhibitor of metalloproteinases (TIMPs), in BALF of ARDS patients. MMP‐2, MMP‐8 and MMP‐9 are proteases secreted by neutrophils and their levels were elevated in all patients together with neutrophil number. Furthermore, elevated MMP‐1 and MMP‐3 were associated with increased mortality 83. Although neutrophils do not produce MMP‐1 and MMP‐3, they can induce MMP‐1 secretion in human vascular smooth muscle cells, which in turn acts in an autocrine feedback loop to produce CXCL8 and induce neutrophil chemotaxis 84. Consistent with this, MMP‐3‐deficient mice have reduced neutrophil migration into the lung and attenuated neutrophil‐mediated epithelial and vascular damage in the context of immune complex‐mediated pulmonary injury 85. MMP‐13 has been shown to play a role in the development of sepsis‐mediated acute lung injury 86. Hypertonic saline has been shown to reduce the production of MMPs, such as MMP‐9 and MMP‐13, in mouse models of acute lung injury, thereby reducing disease progression and inflammation; an open‐label clinical trial is currently underway to evaluate efficacy in post‐traumatic acute lung injury (Table 1) 86, 87, 88.

As mentioned previously, HBP is a cationic peptide that plays an important role in neutrophil‐mediated vascular leakage through increased endothelial permeability 89. In trauma patients admitted to intensive care, early elevation of HBP after admission was a predictor for the development of ARDS, suggesting that HBP may be a potential biomarker for the early detection of ARDS, although further work is required 90. Plasma HBP has also been shown to be an independent predictor for 30‐day mortality in ARDS 91. Administration of simvastatin to patients with acute lung injury reduced HBP plasma levels 92. Simvastatin did not improve overall survival in ARDS patients, but secondary analysis has identified improvement in 28‐ and 90‐day survival in patients with a hyper‐inflammatory subphenotype relative to placebo control (Table 1) 93.

Defensins are arginine‐rich cationic proteins that have antimicrobial properties 94. Divided into two subgroups, α‐defensins and β‐defensins exhibit different roles. Neutrophils store α‐defensins in their granules and release them in an attempt to eradicate microbes, with β‐defensins primarily expressed by mucosal surface epithelial cells. However, defensins can also result in tissue damage, as observed in ARDS 95. Elevated levels of α‐defensins were found in BALF of ARDS patients and higher levels correlate with increased severity of lung injury 95. Although plasma α‐defensin was also elevated it did not correlate with prognosis; it has been proposed that circulating α‐defensin originates from the bone marrow rather than directly from neutrophils and therefore has different functional effects in this context. Although not known to be produced by neutrophils, β‐defensins are implicated in the pathogenesis of ARDS. β‐defensin‐3 inhibits neutrophil apoptosis by downregulating Bid, a pro‐apoptotic protein, and upregulating the anti‐apoptotic protein Bcl‐x_L_ in neutrophils 96. This delay in apoptosis is dependent upon the interaction of β‐defensin‐3 with the chemokine receptor CCR6, with the effect attenuated in the presence of a CCR6‐specific blocking antibody 96. As discussed below, delay in neutrophil apoptosis is associated with an increased severity in lung injury.

LL‐37 is another cationic protein with antimicrobial properties released from neutrophil granules 97. It also carries the ability to activate neutrophils and augment the inflammatory cascade 98 and LL‐37 is elevated in BALF samples of ARDS patients relative to healthy volunteers 97. Interestingly, although elevated LL‐37 correlated with increased lung injury, LL‐37 did not correlate with neutrophil counts, suggesting that neutrophils are not the only source of LL‐37, with macrophages and epithelial cell production also described 97.

## Reactive oxygen species

ROS play an important role in eliminating pathogens within phagosomes and for the generation of NETs, but also act as chemoattractants for immune cells, resulting in tissue repair 99. However, excess ROS production results in oxidative stress and plays a major role in lung damage through the release of pro‐inflammatory cytokines, enhanced recruitment of immune cells and consequently the progression of ARDS 99. Neutrophils have been shown to produce ROS when activated and contribute to oxidative stress 99. Furthermore, increased permeability of the endothelial and epithelial barrier is observed, increasing neutrophil transmigration to the alveolar space 99. Additionally, an increase in oxidised molecules and a reduction in antioxidant proteins are observed in BALF of ARDS patients, which serves to perpetuate lung damage 100. Glutathione plays a vital role in neutralising hydrogen peroxide, a major contributor to oxidative damage, through the enzyme glutathione peroxidase, by converting glutathione to glutathione disulphide 101. Administration of the antioxidant N‐acetylcysteine restores the oxidant balance by increasing glutathione levels in erythrocytes. Several clinical trials have investigated the role of N‐acetylcysteine as a therapeutic strategy in ARDS with variable results. A recent meta‐analysis concluded that, although the duration of intensive care admission is shortened, there is no demonstrable effect on overall outcome or 30‐day survival 102.

## Mechanisms of cell death

In addition to marked inflammatory cell activation and recruitment, the pathogenesis of ARDS is characterised by alterations in a variety of forms of cell death. Death and damage to the alveolar epithelial and alveolar endothelial cells are thought to play a key role in the initiation and progression of the disease process 103, 104, whereas inflammatory cell apoptosis and subsequent clearance is an important step in inflammation resolution 105, 106. Although apoptosis (described further below) is undoubtedly the most studied form of cell death, there has also been a recent ‘‐osis explosion’ with increased knowledge of alternative cell death pathways, such as pyroptosis, necroptosis, ferroptosis, entosis and NETosis 107. Although some of these non‐apoptotic pathways probably have relevance to the pathogenesis of ARDS, this is an as yet understudied area that will hopefully lead to future novel avenues for therapeutic intervention.

## Targeting apoptosis

Apoptosis occurs through two distinct but converging pathways. The intrinsic pathway is activated in response to diverse stimuli, including DNA damage, ROS exposure and endoplasmic reticulum stress. The central event in intrinsic apoptosis is mitochondrial outer membrane permeabilisation that allows escape of pro‐apoptotic molecules such as cytochrome‐c, which then form a caspase‐activating complex. Active caspases act as the executioners of apoptosis, leading to cellular disassembly of the cell, DNA degradation, cell surface phosphatidylserine exposure and pannexin channel activation, all hallmarks of apoptotic cell death. Mitochondrial outer membrane permeabilisation is itself controlled by intracellular Bcl2 family proteins, which include both pro‐ and anti‐apoptotic members (such as Bid and Bcl‐xL, described above). In contrast, extrinsic apoptosis is usually activated by a cell surface death receptor upon interaction with its cognate ligand, which then leads to caspase activation. The multiple steps and checkpoints involved in apoptotic cell death allow this to be dysregulated at multiple steps in human diseases such as ARDS, but also allow the potential for therapeutic intervention at several levels 108.

Neutrophil apoptosis in ARDS has been shown to be delayed by several groups, including our own 109, 110, 111, 112, and correlates with disease severity in sepsis and sepsis‐related ARDS. Interestingly, BALF from patients with early ARDS (days 1 and 3 of disease) but not late ARDS directly inhibits apoptosis of healthy donor neutrophils 109. This effect has been attributed to soluble factors, including GM‐CSF, G‐CSF, CXCL8 and IL‐2 109, 113. Recent detailed characterisation of ARDS neutrophils has revealed multiple phenotypic alterations alongside delayed apoptosis 112. Interestingly, ARDS BALF‐induced delay of healthy neutrophil apoptosis could be overcome by PI3K inhibition, whereas the anti‐apoptosis phenotype of ARDS patient neutrophils was resistant to PI3K inhibition 112. This suggests that additional PI3K‐independent mechanisms are in play within the complex pro‐inflammatory milieu experienced during human ARDS.

Several other preclinical strategies targeting neutrophil apoptosis have also shown promise in the treatment of lung injury. Targeting of the extrinsic pathway of apoptosis has been achieved by TNF‐related apoptosis‐inducing ligand (TRAIL), part of the TNF family of ligands that can initiate apoptosis by activating cell surface receptors 114. TRAIL appears to have no role in constitutive neutrophil apoptosis nor neutrophil chemotaxis (in contrast to the TNF family ligand FasL, which is a potent neutrophil chemoattractant). However, in response to LPS‐induced lung injury, TRAIL acts to limit inflammation and enhances neutrophil apoptosis 115. Furthermore, recombinant TRAIL was able to induce an anti‐inflammatory response, suggesting that such strategies may have therapeutic potential in human ARDS. Targeting of the intrinsic pathway of neutrophil apoptosis, such as with CDK inhibitor drugs, has also been shown to have potent anti‐inflammatory effects in animal models of neutrophil dominant inflammation 14, 15. CDK inhibitor drugs principally induce neutrophil apoptosis by inhibiting CDK9‐mediated transcription of the short‐lived Bcl2 member Mcl‐1 14, 116. As neutrophils have limited expression of the main anti‐apoptotic Bcl2 homologue, Bcl2 itself, this leaves them sensitive to alterations in Mcl‐1, leading to apoptosis. CDK inhibitor drugs enhance the resolution of several lung injury models, including bleomycin‐induced, endotoxin‐induced and bacteria‐induced lung injury 14, 117. Interestingly, in an *Escherichia coli*‐induced model of acute lung injury, a CDK inhibitor drug administered after the onset of inflammation augmented the resolution of lung inflammation without detrimentally reducing clearance of the bacteria. Indeed, there was increased bacterial clearance, possibly resulting from lipid‐mediated enhanced bacterial phagocytosis by macrophages 14. Furthermore, and in contrast to that observed with PI3K inhibition 112, CDK inhibitor has recently been shown to override the delayed neutrophil apoptosis in sepsis‐induced human ARDS concurrent with reduced expression of Mcl‐1 111. This suggests that Mcl‐1 targeting approaches (either with CDK inhibitor or with the use of novel small molecule Mcl‐1 inhibitors) may have therapeutic potential in human ARDS.

Other strategies to modulate neutrophil apoptosis warrant further investigation in the context of ARDS. Potential strategies include the use of a p21 (Cdnk1a) peptide, which binds and sequesters proliferating cell nuclear antigen PCNA, an important endogenous neutrophil anti‐apoptotic factor 118. Although p21 peptide is able to induce apoptosis of neutrophils isolated from patients with lung inflammation 119, testing in *in vivo* models of ARDS is awaited. Several families of anti‐inflammatory lipid mediators that influence neutrophil lifespan and their clearance (among other pleiotropic effects) have also been delineated. These include the lipids 15‐epi‐lipoxin A4 and resolvin E1, which drive neutrophil apoptosis and attenuate experimental lung injury 120, 121.

## Lung parenchymal cell death

In contrast to the potential benefits of inflammatory cell apoptosis during lung injury, there is also evidence that damage and death of the lung epithelium and endothelium can contribute to disease pathogenesis 109. Alveolar epithelial apoptosis has been observed in experimental lung injury caused by bleomycin, endotoxin and acid 31, 122, while alterations in Bcl2 members (including increases in pro‐apoptotic Bax) have been observed in lung epithelium from human lung injury cases 123. In addition, BALF from human ARDS patients contains FasL, which activates Fas receptor to induce extrinsic pathway apoptosis 124. Lung epithelium expresses Fas, with ARDS BALF able to induce epithelial apoptosis in a Fas/FasL‐dependent manner 124, but abolished Fas activity was unable to protect in experimental virus‐induced ARDS 125. Further work is needed to clarify the role and timing of Fas/FasL strategies, especially as Fas can also influence macrophage dynamics during resolution of lung injury 126.

Similarly, death of pulmonary endothelium has recently been demonstrated to be a pathogenic response in endotoxin‐induced lung injury 127. This elegant study demonstrated that endotoxin exposure led to activation of caspase‐4/5 (caspase‐11 in mice) in endothelium and a consequent pro‐inflammatory, lytic form of cell death (termed pyroptosis). Conditional deletion of caspase‐11 specifically in endothelial cells (using a Cre/lox system) led to reduced endotoxin‐induced lung oedema, neutrophil accumulation and death. Caspase‐11 inhibitors, such as wedololactone, suppress endotoxin‐induced caspase‐11 *in vitro* 128, but their role in lung injury *in vivo* remains to be tested. In summary, any potential anti‐inflammatory strategy based on modulation of inflammatory cell death has to carefully balance potentially deleterious off‐target effects should cell death be induced in lung parenchymal cells.

## Clearance of apoptotic cells

Macrophages play a crucial role in limiting excessive inflammation and augmenting tissue repair, not only in the clearance of apoptotic and necrotic cells, but also in the removal of neutrophils undergoing NETosis 129, 130. In ARDS, however, macrophage phagocytic function is impaired 130. Grégoire and colleagues 130 observed enhanced NET formation and reduced neutrophil apoptosis coupled with a reduction in macrophage clearance of apoptotic cells (efferocytosis) in BALF from ARDS patients. AMP‐activated protein kinase (AMPK) has been associated with increasing macrophage phagocytosis and reduced TNF and IL‐6 production 131. The addition of metformin, an AMPK activator, to ARDS BALF samples resulted in removal of NETs and increased efferocytosis by macrophages 130. Additionally, AMPK activators administered in an LPS‐induced mouse lung injury model reduced alveolar neutrophil accumulation, pulmonary oedema and BALF TNF and IL‐6. Furthermore, retrospective analysis of diabetic patients on metformin for the 3 months prior to developing ARDS had a non‐significant reduction in 30‐day mortality from 55.32 to 42.42%. Little is known about the exact anti‐inflammatory mechanism of metformin in this context and therefore it requires further study 132.

A similar observation was made using a neutralising antibody against HMGB1 to increase macrophage efferocytosis 130. HMGB1, which is increased in ARDS 133, inhibits efferocytosis by interfering with the binding between the phosphatidylserine bridging molecule milk fat globule EGF factor 8 (MFG‐E8) and the αvβ3 integrin on the surface of macrophages 134. Importantly, MFG‐E8 knockout mice have increased apoptotic alveolar neutrophils in the alveolar space following LPS‐induced injury, an effect that can be rescued by recombinant MFG‐E8 135.

Another potential mechanism that some have speculated may be involved in neutrophil clearance is the relatively recently described concept of reverse migration. This is the process by which neutrophils migrate in the opposite direction to the chemotactic gradients that initially recruited them 136, 137. As yet, this process has only been demonstrated in zebrafish and mouse models, with no firm data demonstrating a direct role in human disease. In animal models, PGE_2_ is an important mediator of neutrophil reverse migration, with macrophage depletion resulting in inhibited reverse migration and therefore delayed resolution of inflammation due to reduced PGE_2_ production 138. Additionally, PGE_2_ depletion has a similar effect, further validating its importance in resolution. PGE_2_ signals through the EP4 receptor, increasing Alox12 production and consequently lipoxin A_4_, an important pro‐resolving mediator that enhances reverse migration 138. In the context of the resolution of pulmonary inflammation in mice, LTB_4_ released by neutrophils promotes NE release, which subsequently cleaves junctional adhesion molecule‐C (JAM‐C) from the endothelium of post‐capillary venules, facilitating reverse migration of neutrophils 139. Although the departure of neutrophils from sites of inflammation can be considered a sign of inflammatory resolution, reverse migration also has the potential to propagate inflammation. Local ischaemia–reperfusion to the ear skin or cremaster muscle in mice can progress to a systemic inflammatory response in numerous organs, including the lung, heart and liver 139. Pharmacological or genetic interference to either enhance or inhibit reverse migration led to a parallel increase or decrease in secondary inflammation in the lung distant organs 139. In humans, increased levels of soluble JAM‐C were detected in the plasma of ARDS patients, hinting that reverse migration may be occurring, with a significant direct correlation observed between soluble JAM‐C and the severity of multi‐organ failure 139. These data therefore suggest that neutrophil reverse migration may not be simply a clearance mechanism, but has the potential to cause dysregulated systemic pro‐inflammation in ARDS.

## Conclusion

Neutrophils are both a hallmark and a driver of ARDS, acting in concert with other resident and recruited inflammatory cell types to induce a dysregulated, overwhelming and often fatal pro‐inflammatory state within the lung (Figure 1). To conclude that a simple ‘one size fits all’ approach in the context of both the pathogenesis and potential therapeutics is perhaps naive and out‐dated. This conclusion is supported by the identification of distinct subpopulations of ARDS patients who respond differently to fluid management, ventilation strategies and some pharmacological therapies 93, 140, 141. Recognition of these different hypo‐ and hyper‐inflammatory phenotypes within the ARDS cohort, as well as ever‐increasing numbers of predictive and prognostic biomarkers, is leading to a shift in clinical trial design to reduce clinical and biological heterogeneity 3. New combination therapies that target a variety of inflammatory components in ARDS are also being considered to address issues of redundancy, as are cell‐based therapies, including bone marrow‐derived myeloid suppressor cells in infection‐related ARDS 3.

It is therefore essential that variations in neutrophil phenotype and function, as well as understanding of neutrophil behaviour in different patient cohorts, are explored and characterised. As has been outlined, the difficulties of neutrophil‐specific therapies in the context of infection‐related ARDS warrant further exploration but, as in the context of induction of neutrophil apoptosis, this should not stop further investigation, particularly in the context of combination therapies. Further research is also required on the complex chemokine networks, NETosis, mechanisms of inflammation resolution as well as strategies that aim to protect or enhance the repair of damaged epithelial and endothelial beds. It is now over 50 years since ARDS was first described 142 and much has been learnt about its pathogenesis and beneficial supportive ventilation and fluid management strategies but the era of effective pharmacological treatments is yet to dawn. Targeting aspects of neutrophil biology will probably have a place among those therapies.

## Author contributions statement

All authors contributed to manuscript writing and revision. All authors approved the final version.
